# Isolation and Identification of Non-*Saccharomyces* Yeast Producing 2-Phenylethanol and Study of the Ehrlich Pathway and Shikimate Pathway

**DOI:** 10.3390/jof9090878

**Published:** 2023-08-26

**Authors:** Rong Zhou, Qingyi Song, Huili Xia, Na Song, Qiao Yang, Xiaoling Zhang, Lan Yao, Shihui Yang, Jun Dai, Xiong Chen

**Affiliations:** 1 Cooperative Innovation Center of Industrial Fermentation (Ministry of Education & Hubei Province), Key Laboratory of Fermentation Engineering (Ministry of Education), National “111” Center for Cellular Regulation and Molecular Pharmaceutics, College of Bioengineering, Hubei Key Laboratory of Industrial Microbiology, Hubei University of Technology, Wuhan 430068, China; 502000004@hbut.edu.cn (R.Z.); fudong9528@163.com (Q.S.); hhxyxhl@163.com (H.X.); nasong@hbut.edu.cn (N.S.); yaolislan1982@aliyun.com (L.Y.); 2 ABI Group, Donghai Laboratory, College of Marine Science and Technology, Zhejiang Ocean University, Zhoushan 316022, China; qiaoyang1979@whu.edu.cn (Q.Y.); zhangxiaoling@zjou.edu.cn (X.Z.); 3 State Key Laboratory of Biocatalysis and Enzyme Engineering, School of Life Sciences, Hubei University, Wuhan 430062, China; shihui.yang@hubu.edu.cn; 4 College of Bioengineering and Food, Hubei University of Technology, No. 28, Nanli Road, Hongshan District, Wuhan 430068, China

**Keywords:** *Starmerella bacillaris*, 2-PE, Ehrlich pathway, qRT-PCR, whole-genome analysis

## Abstract

2-phenylethanol (2-PE) has been widely utilized as an aromatic additive in various industries, including cosmetics, beer, olive oil, tea, and coffee, due to its rose-honey-like aroma. However, no reports have investigated the production of 2-PE by *Starmerella bacillaris*. Here, *S. bacillaris* (syn., *Candida zemplinina*, and named strain R5) was identified by analysis of morphology, physiology and biochemistry, and 26S rRNA and ITS gene sequence. Then, based on the analysis of whole-genome sequencing and comparison with the KEGG database, it was inferred that strain R5 could synthesize 2-PE from L-phe or glucose through the Ehrlich pathway or shikimate pathway. For further verification of the 2-PE synthesis pathway, strain R5 was cultured in M3 (NH_4_^+^), M3 (NH_4_^+^ + Phe), and M3 (Phe) medium. In M3 (Phe) medium, the maximum concentration of 2-PE reached 1.28 g/L, which was 16-fold and 2.29-fold higher than that in M3 (NH_4_^+^) and M3 (Phe + NH_4_^+^) media, respectively. These results indicated that 2-PE could be synthesized by strain R5 through the shikimate pathway or Ehrlich pathway, and the biotransformation from L-phe to 2-PE was more efficient than that from glucose. The qRT-PCR results suggested that compared to M3 (Phe + NH_4_^+^) medium, the mRNA expression levels of *YAT* were 124-fold and 86-fold higher in M3 (Phe) and M3 (NH_4_^+^) media, respectively, indicating that the transport of L-phe was inhibited when both NH_4_^+^ and Phe were present in the medium. In the M3 (Phe) and M3 (Phe + NH_4_^+^) media, the mRNA expression level of *ADH5* was higher than *PDC*, *hisC*, *GOT1*, and *YAT*, and it was 2.6 times higher and 2.48 times higher, respectively, compared to the M3 (NH_4_^+^) medium, revealing that the key gene catalyzing the dehydrogenation of benzaldehyde to 2-PE is *ADH5*. Furthermore, strain R5 exhibits tolerance to high concentrations of 2-PE, reaching 3 g/L, which conferred an ideal tolerance to 2-PE. In summary, the synthesis pathway of 2-PE, mainly for the Ehrlich pathway, was proved for the first time in *S. bacillaris*, which had not been previously explored and provided a basis for non-*Saccharomyces* yeast-producing 2-PE and its applications.

## 1. Introduction

Recently, the breeding and application of nonconventional yeasts have attracted widespread attention. Nonconventional yeasts include all yeast species, except for *Saccharomyces* yeast and *Schizosaccharomyces*, which are extensively distributed in diverse ecological environments [1]. Consequently, analyzing the biological characteristics of nonconventional yeasts is beneficial for further exploring their application potential in various biotechnology fields.

*Starmerella bacillaris*, one of the species of the genus *Starmerella*, isolated from the fermentation of *Botrytis*-influenced sweet wine in Napa Valley (California, USA) in 2002 [2], initially named *Candida zemplinina* in 2003 [3]. Due to the obvious difference in rRNA sequence compared to *Candida stellata*, *C. zemplinina* was renamed *S. bacillaris* in 2012 [4]. *S. bacillaris* is a non-*saccharomyces* and nonconventional wine yeast, generally distributed on the surface of grapes and winemaking equipment [5]. The yeast is known for its unique properties [6,7], such as strong fructophilic nature, tolerance to low temperature, high glucose concentration [8], and high glycerol concentration [9]. Furthermore, studies have demonstrated that the yeast has the ability to produce mannoproteins characterized by hydrophobicity, high glycosylation, and high molecular weight, which contribute to bubble stabilization and wine quality enhancement [10,11,12].

Non-*Saccharomyces* yeast can promote the production of alcohol, glycerol, volatile aroma substances, mannoproteins, polysaccharides, anthocyanins, and other constituents in wine through various metabolic pathways, thereby affecting the color, flavor, and taste of wine [13]. Among these compounds, 2-phenylethanol (2-PE) conveys a rose-honey-like aroma [14], hence its widespread use as an aromatic additive in many industries, such as cosmetics, beer, olive oil, tea, and coffee [15,16]. In addition, 2-PE exhibits antibacterial properties against the germination and growth of *Penicillium* conidia in a reversible, non-fatal, and concentration-dependent manner [17]. Studies have proved that the enrichment of higher alcohols in wine is mainly attributed to the contribution of the key aromatic alcohol (2-PE) [18,19,20]. Yeasts exhibit the highest efficiency in producing 2-PE among all microorganisms, including *Saccharomyces cerevisiae* (0.68 mg/L) [21], *Kluyveromyces marxianus* (1.94 g/L) [22], *Pichia fermentans* (3.28 g/L) [23], *Candida glycerinogenes* (0.71 g/g L-phe) [24], and *Zygosaccharomyces rouxii* (3.58 g/L) [25].

However, no reports have investigated the production of 2-PE by *S. bacillaris*. Therefore, this study aimed to explore the relationship between *S. bacillaris* and 2-PE, as well as the synthesis pathways of 2-PE in *S. bacillaris*. These findings lay a solid foundation for further research on the application of *S. bacillaris* in various aromatic products, particularly in the wine industry.

## 2. Materials and Methods

### 2.1. Isolation of Yeast Strains

Initially, 10 g of pear peel were weighed and crushed, then added to a 90 mL solution of 0.85% (*w*/*v*) NaCl. The mixture was shaken for 30 min to obtain a microbial suspension. Subsequently, the suspension was diluted by 10-fold serial dilution to 10-2, 10-3, 10-4, 10-5, 10-6, and 10-7 with sterile water. Next, 0.2 mL of each diluted microbial suspension was evenly spread on YEPD plates containing yeast extract (10 g/L), peptone (20 g/L), glucose (20 g/L), and agar (15 g/L), followed by incubation at 28 °C for 24 h. Single colonies were then selected and streaked onto high-glucose (600 g/L) YEPD medium and incubated at 28 °C for 24 h [26]. Finally, the colonies were preserved at −80 °C using a glycerin storage method.

### 2.2. Screening of Yeast Strains Producing 2-PE

The isolated strains were cultured in YEPD broths at 28 °C and 200 r/min for 12 h as seed cultures. The seed cultures were then inoculated into YEPD broths at a concentration of 5% (*v*/*v*) and incubated at 28 °C for 24 h. Subsequently, the fermentation broths were preliminarily judged for an aromatic smell through sensory analysis. Sensory evaluation was performed using a trained panel of 10 experts. The intensity of the flavor attribute, characterized by a rose-honey-like aroma, was quantified on a scale from 0 to 10, where 0 indicated absence and 10 denoted the highest intensity [27,28]. Each sample underwent triplicate assessment, and the mean of each sample was calculated as the average of the three scores on a ten-point scale. The fermentation broths with the highest score were centrifuged at 12,000 r/min for 10 min to collect the supernatant. Then, 10 mL of supernatant was sealed in a headspace bottle and equilibrated at 40 °C for 20 min. Finally, the volatile flavor compounds in the supernatants were collected using an extraction fiber for 20 min and analyzed using an Agilent 7890B GC coupled with an Agilent 5977B MS (GC-MS, Agilent Technologies, Santa Clara, CA, USA) [29] to identify strains producing 2-PE. The aforementioned experiments were repeated three times. The strains that produced 2-PE were selected as the target strains for subsequent experiments.

GC-MS parameters were set according to Zhou et al. [30]: The injector temperature was maintained at 260 °C. Chromatographic separations were performed using a DB-Wax column (30 m × 0.25 mm ID, 0.25 m film thickness). Helium was employed as the carrier gas with a constant flow rate of 0.8 mL/min. The oven temperature program was initially held at 40 °C for 4 min, then raised to 160 °C at a rate of 6 °C, and subsequently increased at a rate of 10 °C/min to 220 °C for 6 min. Electron impact ionization was performed with an electron energy of 70 eV, and the mass range was set to *m*/*z* 50–550. Identification of each peak was carried out by comparison with NIST 11 library (Agilent Technologies).

### 2.3. Identification of Yeast Strains Producing 2-PE

The morphology, physiology, and molecular biology of yeast strains that produce 2-PE were characterized.

#### 2.3.1. Observation of Morphology

The colony morphology of strains cultured on YEPD plates at 28 °C for 2–4 days [31] was observed, including colony color, shape, surface folds, transparency, and size. The sediment amount, as well as the presence of rings or islands, was inspected in YEPD broths at 28 °C for 24 h [32]. Additionally, the cell morphology and asexual reproduction mode of the cultured strains were examined using a Zeiss microscope (Scope. A1, Carl Zeiss AG, Jena, Germany).

#### 2.3.2. Physiological Characteristics

The physiological and biochemical characteristics were determined using Vitek 2 compact 60 system [33,34] in this work.

The experiment using the Vitek 2 compact 60 system was conducted following the workflow below: (1) Single colony of strains was uniformly dispersed in a turbidimetric glass tube containing 3 mL of 0.85% (*w*/*v*) NaCl solution. (2) The McFarland turbidity of the turbidimetric glass tube was adjusted to 0.44~0.56 using 0.85% (*w*/*v*) NaCl solution at a wavelength of 530 nm, measured with the DensiChek instrument (BioMerieux, Marcy-l’Étoile, France). (3) The Gram-positive identification card was inserted into the yeast liquid tube and then placed in the automatic cell identification system of Vitek 2 compact 60 system.

#### 2.3.3. Molecular Biological Identification

Genomic DNA was extracted from a fresh culture of yeast strains followed by TIANamp Yeast DNA Kit (Tiangen Biotech (Beijing) Co., Ltd., Beijing, China) [35,36] for sequencing and phylogenetic analysis of the 26S rRNA gene and ITS gene. Primers 26S-F (5′-GCATATCAATAAGCGGAGGAAAAG-3′) and 26S-R (5′-GGTCCGTGTTTCAAGACGG-3′) were used to amplify the D1/D2 region [37]. Primers ITS1 (5′-TCCGTAGGTGAACCTGCGG-3′) and ITS4 (5′-TCCTCCGCTTATTGATAT GC-3′) were used to amplify the ITS gene region [38]. The amplification protocol [7] consisted of initial denaturation at 95 °C for 5 min, followed by 31 cycles of amplification at 94 °C for 3 s, 92 °C for 30 s, 40 °C for 1 min, and a final extension at 65 °C for 8 min.

Then amplification products were sequenced by Qingke Biotechnology Co., Ltd., Beijing, China. Sequence similarity was determined using NCBI BLAST (National Center for Biotechnology Information, Bethesda, MD, USA) based on databases—Nucleotide collection (nr/nt), employing Megablast for highly similar sequences. The phylogenetic analysis was conducted by using MEG A-X(64) software after multiple alignments of data via CLUSTAL_X, and the phylogenetic tree was constructed using the neighbor-joining method [39].

### 2.4. Whole-Genome Sequencing, Assembly, and Annotation of Strain R5

#### 2.4.1. Genomic DNA Extraction and Sequencing

Yeast strains were cultured in YEPD medium at 28 °C for 24 h. Subsequently, 1 mL of cultures was centrifuged at 12,000 rpm and 4 °C for 1 min to separate the supernatant from the cells, which were then harvested. Yeast cell DNA was extracted using the TIANamp Yeast DNA Kit (Tiangen Biotech (Beijing) Co., Ltd.) [35,36] and subsequently sequenced by Grandomics Biosciences Co., Ltd., Beijing, China.

#### 2.4.2. Whole-Genome Data Processing and Analysis

DNA samples were qualified by library preparation and high-throughput sequencing (HTS) to acquire the respective nucleotide sequence data.

Library preparation and high-throughput sequencing (HTS) were performed on qualified DNA samples to obtain corresponding nucleotide sequence data. Genome size and genome heterozygosity were evaluated based on the sequence file after quality control and the software FindGSE (v1.94.R) and GenomeScope (2.0.1) corresponding to the two algorithms. Subsequently, the final results of genome evaluation were generated. The final assembly results were compared and analyzed with databases such as GO, KEGG, and NR to achieve gene annotation information prediction and function prediction [40,41].

### 2.5. Study of the Synthesis Pathway of 2-PE

To investigate the synthesis pathway of 2-PE, strain R5 was inoculated into three M3 media at a concentration of 5% (*v*/*v*) at 28 °C for 72 h. The OD_600_ and content of glucose, L-phe, and 2-PE in three M3 media based on Mierzejewska [42] were detected during the process of fermentation. The specific components of the three M3 media (Table 1) were as follows:

The composition of M3 (Phe) medium comprised 30 g/L glucose, 8 g/L sucrose, 1.7 g/L YNB, 9 g/L L-phe, and 0.5 g/L MgSO_4_·7H_2_O; M3 (Phe + NH_4_^+^) medium was made from 30 g/L glucose, 8 g/L sucrose, 1.7 g/L YNB, 4.5 g/L L-phe, 2.25 g/L (NH4)_2_SO_4_, and 0.5 g/L MgSO_4_·7H_2_O; the components of M3 (NH_4_^+^) medium were as follows: 30 g/L glucose, 8 g/L sucrose, 1.7 g/L YNB, 4.5 g/L (NH4)_2_SO_4_, and 0.5 g/L MgSO_4_·7H_2_O.

Initially, 5 mL fermentation broths were collected every 10 h by centrifugation at 10,000 rpm for 5 min at 4 °C. The supernatants were subsequently analyzed for glucose concentration using the DNS method [43], while 2-PE and L-phe were quantified using high-performance liquid chromatography (HPLC) with a C-18 column (ThermoFisher Scientific, San Diego, CA, USA). A solvent consisting of ultra-pure water/methanol (40/60) or water/methanol (50/50) was used as the mobile phase for 2-PE or L-phe, respectively, at a constant flow rate of 0.6 mL/min or 1 mL/min. Finally, the concentrations of 2-PE and L -Phe were detected at 210 nm and 260 nm, respectively.

### 2.6. Quantitative Real-Time PCR (qRT-PCR)

The total RNA of yeast strains cultivated in three M3 media for 12 h was extracted using the RNA simple Total RNA kit (Tiangen Biotech (Beijing) Co., Ltd.), following the manufacturer’s instructions. Subsequently, DNA elimination and reverse transcription were performed using the HiScript II QRT SuperMix (Vazyme Biotech Co., Ltd., Nanjing, China). Then, PCR was quantified on the CFX96TM RT-PCR system (Bio-Rad, Hercules, CA, USA) after adding the ChamQ Universal SYBR qPCR Master Premix (Vazyme Biotech Co., Ltd.). Finally, the relative expression levels of mRNA were determined using the 2^−∆∆CT^ method [44]. Among them, ΔCTs were derived by the CTs (cycle thresholds) of the target genes (*YAT*, *GOT1*, *his C*, *PDC*, and *ADH5*) minus the CT of *ENO1*, which served as the housekeeping gene. ΔΔCTs were calculated by ΔCTs from the target genes minus the CT of the control gene. Fold changes were determined using the 2^−∆∆CT^ method. The primers of related genes are listed in Table 2.

### 2.7. Tolerance of Yeast Strains to 2-PE

Yeast strains were inoculated into YEPD medium at a concentration of 5% (*v*/*v*) and cultured at 28 °C with agitation at 200 rpm. Once the OD_600_ reached 0.8, 2-PE was added successively into YEPD medium to obtain concentrations of 0, 1, 2, 3, 4, and 5 g/L [45]. The YEPD medium without 2-PE served as a blank control. Each YEPD medium with varying 2-PE concentrations was cultured at 28 °C for 200 rpm, and triplicate biological replicates were prepared for each concentration. The tolerance to 2-PE of yeast was determined by measuring the OD_600_, which was monitored at 12 h intervals until it reached a stable value.

## 3. Results and Discussion

### 3.1. Isolation and Screening of Yeast Strains Producing 2-PE

**Figure 1:**
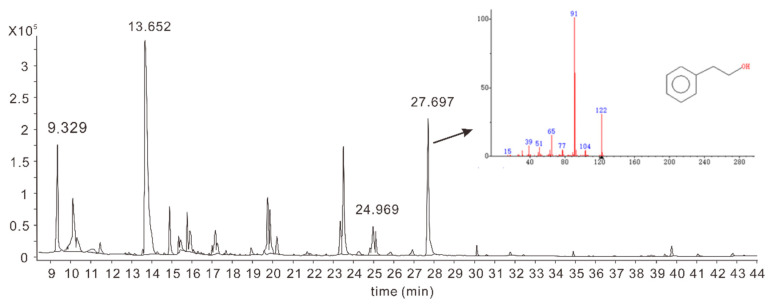
Volatile compounds in stationary phase of fermentation produced by strain R5.

Four strains grew rapidly in the high-glucose YEPD medium. Among them, 2-PE was found in a strain with the highest intensity of rose-honey-like aroma, while it was not detected in the other three yeast strains. This strain, named R5, was chosen as the target strain for subsequent experiments. 2-PE accounted for 10.2% of the total flavor components in the supernatant of strain R5, as depicted in Figure 1.

### 3.2. Identification of Morphology, Physiology, and Molecular Biology

**Figure 2:**
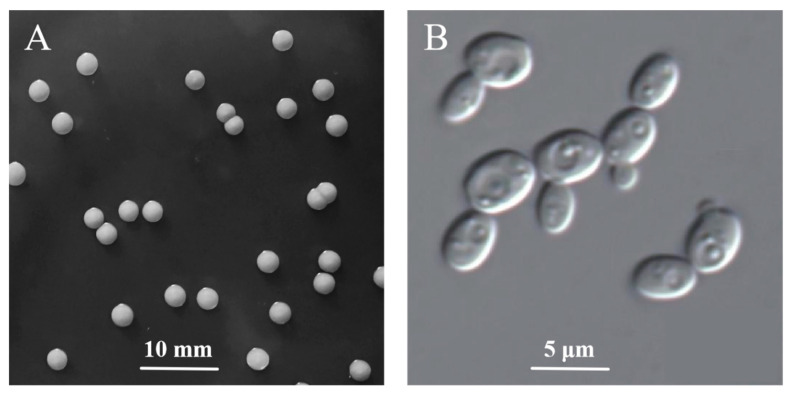
Colony morphology (**A**) and cell morphology (**B**) of strain R5.

As shown in Figure 2A, the colony morphology of strain R5 exhibited a milky appearance, characterized by a circular or oval shape with well-defined edges and a smooth, opaque surface. Colonies with a diameter ranging from 2 to 4 mm were loosely attached to the medium and could be easily picked. No sediment, rings, or islands of strain R5 were observed in YEPD broths. In Figure 2B, strain R5 exhibited a colorless and transparent spherical or ellipsoidal shape with a cell diameter in the range of 2.5–3.3 × 4.0–5.0 µm, which was smaller than the cell size of common strains of *Saccharomyces* strains (5–10 µm). Strain R5 cells reproduced through budding (asexual reproduction). Mature mother cells developed bud cells at specific sites, and the bud cells detached from the mother cells, producing new individual cells.

Table 3 displays the results of the physiological and biochemical identification for strain R5. The results were compared with the standard strains in the “Manual of Yeast Characteristics and Identification” [46], revealing that strain R5 was preliminarily identified as *S. bacillaris* [3].

**Figure 3:**
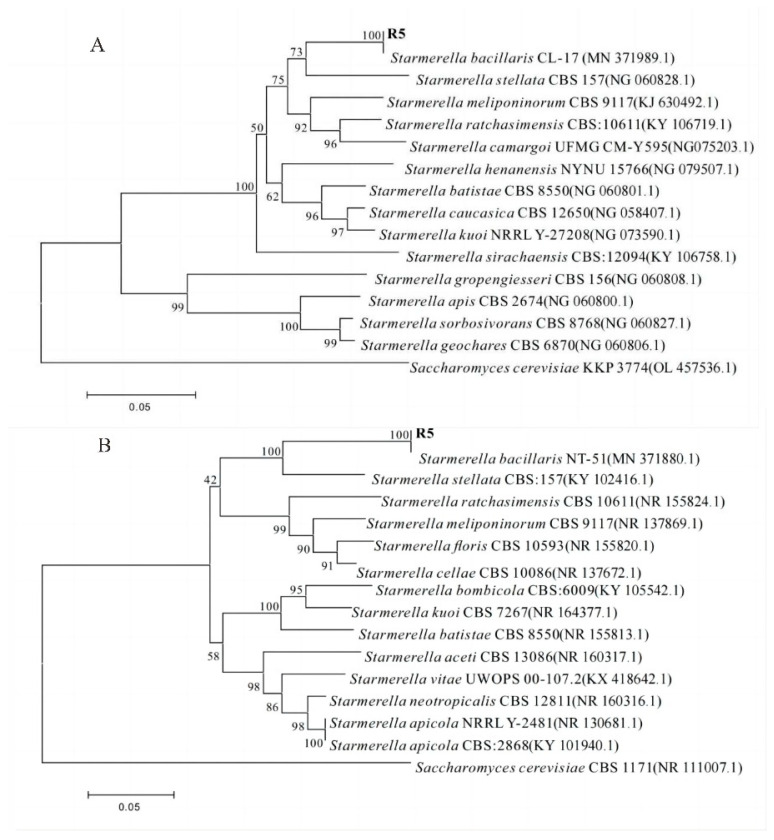
Phylogenetic trees based on of 26S rRNA sequences (**A**) and ITS gene sequences (**B**).

Phylogenetic trees (Figure 3) were constructed to elucidate the phylogenetic relationship between the isolate and other species of the genus *Starmerella*. The support for this relationship came from the dendrogram of the 26S rRNA gene sequence and ITS gene sequence. Based on comparative analysis of the 26S rRNA gene sequence (Figure 3A), strain R5 was found to be closely related to members of the genus *Starmerella*, specifically forming a distinct branch with *S. bacillaris* CL-17 (MN 371989.1). Strain R5 exhibited sequence similarity values below 97.0% with recognized members of the genus *Starmerella*, including *S. ratchasimensis* CBS 10611 (KY 106719.1), *S. kuoi* NRRL Y-27208 (NG 073590.1), *S. sorbosivorans* CBS 8768 (NG 060827.1), *S. gropengiesseri* CBS 156 (NG 060808.1), and *S. geochares* CBS 6870 (NG 060806.1). ITS gene sequence comparison (Figure 3B) confirmed that strain R5 resided within the genus *Starmerella*, forming a distinct branch alongside *S. bacillaris* NT-51 (MN 371880.1). Compared with recognized members of the genus *Starmerella*, including *S. apicola* NRRL Y-2481 (NR 130681.1), *S. apicola* CBS: 2868 (KY 101940.1), *S. ratchasimensis* CBS 10611 (NR 155824.1), *S. kuoi* CBS 7267 (NR 164377.1), and *S. bombicola* CBS: 6009 (KY 105542.1), the sequence similarity values were all below 97.0%. The findings from the analysis of 26S rRNA and ITS were in line with the research conducted by Goncalves [47]. Based on the aforementioned results, strain R5 is likely to belong to the *bacillaris* species within the genus *Starmerella*. The sequence similarity values between strain R5 as well as *S. bacillaris* CL-17 (MN 371989.1) (Figure 3A) and *S. bacillaris* NT-51 (MN 371880.1) (Figure 3B) were 99%. Thus, the observed branching pattern and similarity results strongly support the identification of strain R5 as *S. bacillaris* [48].

### 3.3. Whole-Genome Sequencing, Assembly, and Annotation of Strain R5

The genome of strain R5 was assembled using PacBio single-molecule real-time (SMRT) sequencing, which provided over 200-fold coverage of the genome. A hierarchical assembly strategy followed by scaffolding yielded 250 scaffolds, totaling 5.70 Gb in size, which were subsequently placed into chromosomal scaffolds based on homology to *S. cerevisiae* S288C. The N90 length and N50 length were 1.1 Mb and 3.81 Mb, respectively, as shown in Table 4. Based on the k-mer analysis of strain R5, the genome size was estimated to be approximately 9.29–10.41 Mb with a heterozygosity rate of 0.09%.

The gene density and average GC content in the genome of strain R5 were similar to those reported for other hemiascomycetous yeasts (Table 5), including *S. bacillaris* PAS13 [11] and *S. cerevisiae* S288c [44]. The genome of strain R5 was predicted to contain a total of 3991 genes, which was also approximately similar to the annotated genes of *S. bacillaris* PAS13 (4321 genes). Moreover, the total genome size of strain R5 (9.47 Mb) closely matched the haploid genome size of the reference strain *S. bacillaris* PAS13 (9.4 Mb). These findings suggest that the genome of R5 is likely haploid.

Based on the KEGG database analysis, the metabolic pathway exhibited the highest number of genes assigned to carbohydrate metabolism (141 genes), followed by amino acid metabolism (141) and cofactor/vitamin metabolism (131). Additionally, these genes were enriched in molecular function (1692), metabolic process (7686), and cellular component (21,714), resulting in the definition of 9 gene categories based on molecular function, 16 gene categories based on metabolic process, and 10 gene categories based on a cellular component.

**Figure 4:**
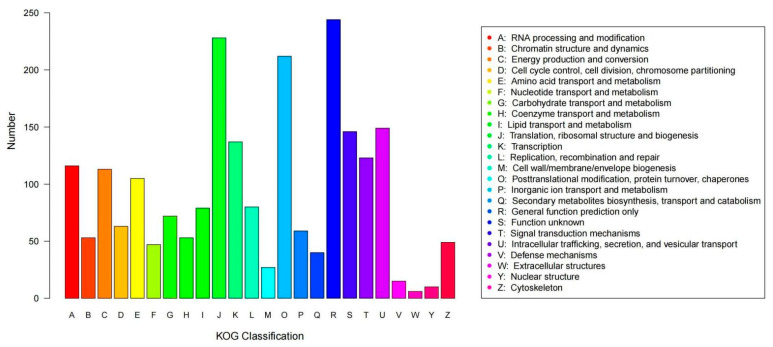
KOG function categories of strain R5. Note: the genome protein sequences of strain R5 were compared with the KOG database by Blastp software, and its functions annotated by protein sequences were sorted.

The predicted genes of strain R5 were functionally annotated using KOG (EuKaryotic Orthologous Groups of protein) function categories. Figure 4 shows that the highest number of genes (244 genes) was assigned to “general function prediction only”, followed by “translation, ribosomal structure and biogenesis” (228 genes), and “posttranslational modification, protein turnover, chaperones” (212 genes).

### 3.4. Identification Synthesis Pathway of 2-PE by Strain R5

**Figure 5:**
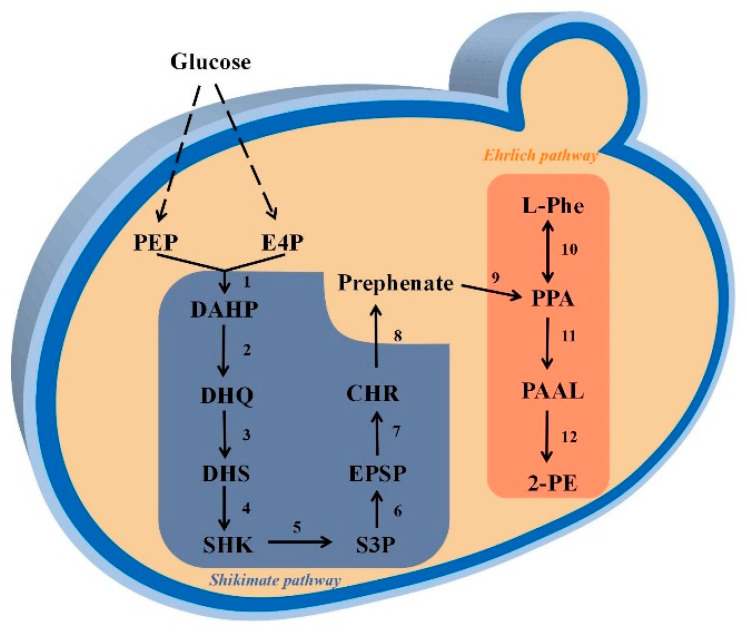
Prediction for 2-PE synthesis pathway of strain R5. Note: PEP: phosphoenolpyruvate, E4P: erythrose-4-phosphate, DAHP: 3-deoxy-d-arabinoheptanose-7-phosphate, DHQ: 3-dehydroquinic acid, DHS: 3-dehydroshikimic acid, SHK: shikimate, S3P: shikimate-3-phosphate, EPSP: 5-enolpyruvate 3-phosphate, CHR: chorismite, PPA: phenylpyruvate, PAAL: phenylacetaldehyde, 2-PE: 2-phenyl alcohol, L-phe: L-phenylalanine.

The KEGG pathway analysis (Figure 5 and Table S1) revealed that Strain R5 possesses both the shikimate pathway and the Ehrlich pathway [49]. Glucose serves as the precursor for 2-phenylethanol (2-PE) production, with phosphoenolpyruvate (PEP) and erythrose-4-phosphate (E4P) being synthesized via the glycolysis pathway and the pentose phosphate pathway, respectively. Subsequently, 3-deoxy-D-arabinoheptanose-7-phosphate synthase, encoded by *aroG* and *aroA* (Contig 1.1159), catalyzes the formation of 3-deoxy-d-arabinoheptanose-7-phosphate (DAHP) from these compounds. Following a series of reactions, DAHP is further converted to shikimate (SHK) through the activities of 3-dehydroquinic acid synthase, 3-dehydroquinic acid dehydratase, and shikimate dehydrogenase. Shikimate kinase, encoded by *Aro1* (Contig 2.434), phosphorylates SHK to generate shikimate-3-phosphate (S3P). Enzymatic reactions catalyzed by 5-enolpyruvylshikimate-3-phosphate synthase and chorismate synthase convert S3P to chorismate (CHR). Chorismate mutase facilitates the conversion of CHR to benzoate. Then, pyrophosphate thiamine-dependent decarboxylase, encoded by *PDC* (Contig 2.377), catalyzes the transformation of phenylpyruvate (PPA) into phenylacetaldehyde (PAAL). PPA also has the potential to be converted to phenylalanine (Phe) or tyrosine by an aminotransferase. The hydrogenation of PAAL to synthesize 2-PE involves the participation of dehydrogenases encoded by *frmA*, *ADH5*, and *adhC* (Contig 2.542) (Table S1) [50]. Alternatively, in the Ehrlich pathway, 2-PE is produced through the biotransformation of L-phe as the precursor. The synthesis of 2-PE in this pathway involves three genes: *GOT1*, *PDC*, and *ADH5*. Specifically, *GOT1* (Contig 2.1565) is responsible for the biosynthesis of aspartate aminotransferase, while the *YAT* gene is believed to play a role in the transport of L-phe.

**Figure 6:**
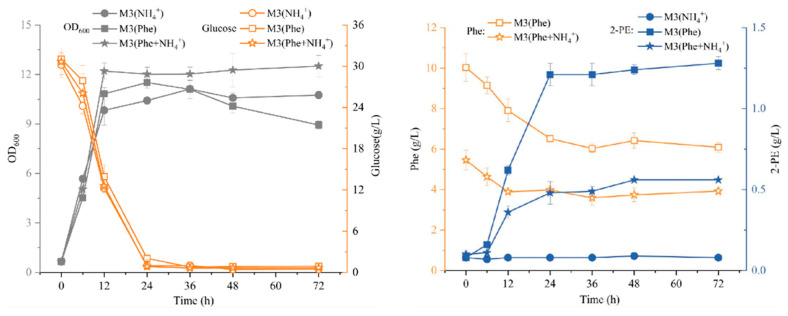
Effect of different nitrogen sources on the growth and 2-PE synthesis of strain R5.

To further investigate the synthesis pathway of 2-PE, strain R5 was inoculated into three different media: M3 (Phe), M3 (NH_4_^+^), and M3 (Phe + NH_4_^+^). L-phe or (NH_4_)_2_SO_4_ was used as the sole nitrogen source in M3 (Phe) or M3 (NH_4_^+^) media, respectively. When strain R5 was cultured in M3 (Phe) medium, 2-PE was biosynthesized from L-phe via the Ehrlich pathway. When strain R5 was inoculated in M3 (NH_4_^+^) medium, 2-PE was produced from glucose through the shikimate pathway [51,52]. As presented in Figure 6, strain R5 reached the stationary phase in M3 (Phe) and M3 (NH_4_^+^) media after 12 h, with OD_600_ values of 10.83 and 9.83, respectively. This suggests that the growth of strain R5 was similar in M3 (Phe) and M3 (NH_4_^+^) media. However, the maximum concentrations of 2-PE in M3 (NH_4_^+^) and M3 (Phe + NH_4_^+^) media were 0.08 g/L and 0.56 g/L, respectively. In M3 (Phe) medium, the maximum concentration of 2-PE reached 1.28 g/L, which was 16-fold and 2.29-fold higher than that in M3 (NH_4_^+^) and M3 (Phe + NH_4_^+^) media, respectively. Moreover, the productivity of 2-PE in M3 (Phe) and M3 (Phe + NH_4_^+^) media was 50.4 mg/L/h and 20 mg/L/h, respectively, which was significantly higher than that in M3 (NH_4_^+^) medium. Additionally, in M3 (Phe) and M3 (Phe + NH_4_^+^) media, L-phe consumption amounted to 3.51 g/L and 1.48 g/L, respectively, at rates of 146 mg/L/h and 61.7 mg/L/h. These findings suggest that strain R5 is capable of synthesizing 2-PE from both L-phe and glucose through the Ehrlich pathway and shikimate pathway, with the biotransformation from L-phe to 2-PE being more efficient than that from glucose.

Moreover, the production of 2-PE in M3 (Phe) and M3 (Phe + NH_4_^+^) media was below the maximum theoretical concentration. This result suggests that L-phe can support growth apart from the biotransformation of 2-PE via the Ehrlich pathway. Further investigation is needed to assess this, including determining the concentrations of phenylpyruvate and PAAL, as well as the activities of related enzymes in future studies. Additionally, the conversion rate of 2-PE in the *S. cerevisiae* S288c strain overexpressing *ARO8* and *ARO10* was reported as 0.5 mol/mol [44]. In comparison, the R5 strain exhibited conversion rates of 0.49 mol/mol and 0.44 mol/mol in M3 (Phe + NH_4_^+^) and M3 (Phe) media, respectively, indicating clear advantages in 2-PE production.

### 3.5. Identification of Potential Genes Involved in 2-PE Synthesis by qRT-PCR

Based on the KEGG database, *YAT* (encoding yeast amino acid transporter), *GOT1* (encoding aspartate aminotransferase, cytoplasmic), *hisC* (encoding aminotransferase), *PDC* (encoding pyruvate decarboxylase), and *ADH5* (encoding S-(hydroxymethyl)glutathione dehydrogenase/alcohol dehydrogenase) were annotated and functionally predicted. The *GOT1*, *hisC*, and *PDC* genes were implicated in the Ehrlich pathway, which is considered efficient for producing 2-PE in strain R5. To further identify potential genes associated with the Ehrlich pathway, the expression levels of the aforementioned genes were measured using qRT-PCR.

**Figure 7:**
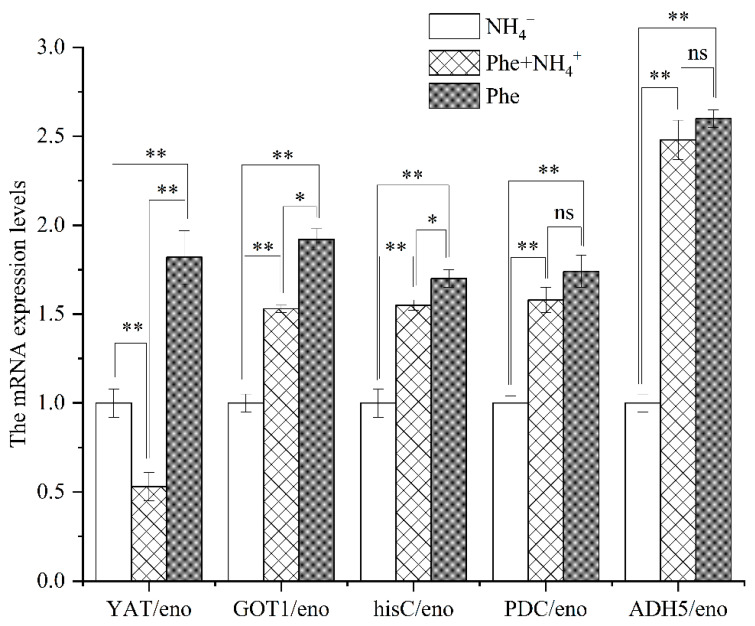
mRNA expression levels of key genes of strain R5 in different nitrogen sources. Note: * represents *p* < 0.05; ** represents *p* < 0.01, and ns represents no significance.

The qRT-PCR results are presented in Figure 7. Compared to M3 (Phe + NH_4_^+^) media, the mRNA expression levels of *YAT* were 124-fold and 86-fold higher in M3 (Phe) and M3 (NH_4_^+^) media, respectively, indicating that the transport of L-phe was inhibited when both NH_4_^+^ and Phe were present in the medium. In the M3 (Phe) and M3 (Phe + NH_4_^+^) media, the mRNA expression level of *ADH5* was higher than *PDC*, *hisC*, *GOT1*, and *YAT*, and it was 2.6 times higher and 2.48 times higher, respectively, for the M3 (NH_4_^+^) media, revealing that the key gene catalyzing the dehydrogenation of benzaldehyde to 2-PE is *ADH5*. Meanwhile, the mRNA expression levels of *ADH5*, *PDC*, *hisC*, and *GOT1* in the M3 (Phe) and M3 (Phe + NH_4_^+^) media were similar to or higher than that in M3 (NH_4_^+^) media. These results demonstrate that NH_4_^+^ has little effect on the expression levels of *ADH5*, *PDC*, *hisC*, and *GOT1*, which are not sensitive genes to nitrogen metabolism inhibition. These findings provide evidence that strain R5 is capable of biotransforming L-phe into 2-PE through an alternative pathway. However, further research is required to validate this hypothesis.

### 3.6. Effect of 2-PE Stress on the Growth of R5 Strain

**Figure 8:**
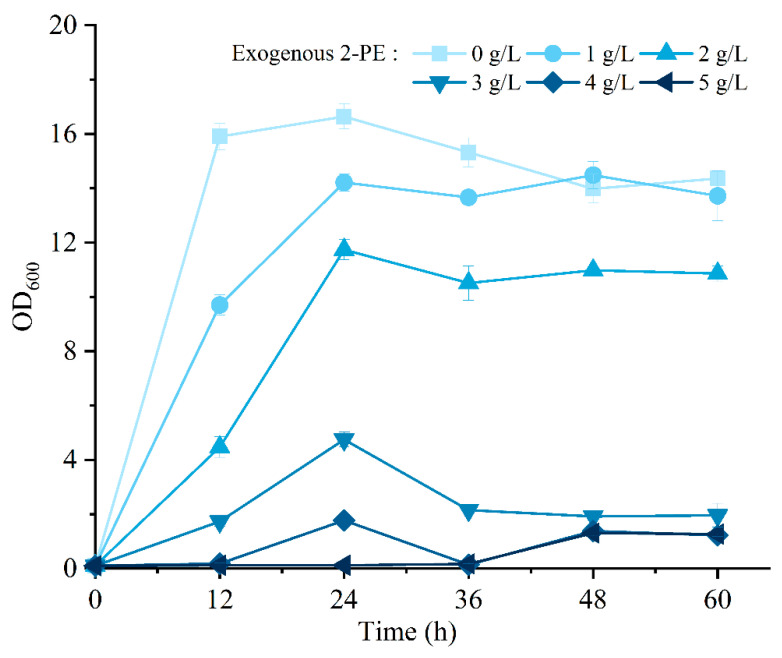
Effect of exogenous 2-PE on strain R5 growth.

Previous studies have provided evidence of the considerable cellular toxicity associated with 2-PE, resulting in the accumulation of reactive oxygen species (ROS), lipid peroxidation, and damage to the cell membrane. These effects have been found to significantly inhibit 2-PE production [45,53,54,55]. However, the toxicity of 2-PE varies among yeast species and is dependent on strain. For instance, the growth of *K. marxianus* CBS 600 was completely inhibited when exposed to 2.6 g/L of 2-PE [49]. A similar study focusing on *K. marxianus* CCT 7735 revealed that the strain growth was limited by 64% at a concentration of 2.5 g/L of 2-PE [56]. Haploid yeast BY2M has demonstrated lower tolerance to 2-PE compared to diploid or wild-type strains [16], as evidenced by the severe growth inhibition observed with the addition of 1.5 g/L 2-PE [21]. To investigate the tolerance of strain R5 towards 2-PE, exogenous 2-PE was supplemented in YEPD medium at final concentrations of 1 g/L, 2 g/L, 3 g/L, 4 g/L, and 5 g/L. Figure 8 demonstrates that at a concentration of 2 g/L, the OD_600_ of strain R5 was 10.86, which was decreased by 24.37% compared with that without 2-PE. When the concentration of 2-PE reached 3.0 g/L, the OD_600_ exhibited a significant decrease of 86.35% compared with the blank control. Nevertheless, the growth of strain R5 was almost completely inhibited when the concentration of 2-PE exceeded 4 g/L. The above results show that strain R5 was tolerant to 2-PE up to a concentration of 3 g/L, which conferred higher tolerance to 2-PE compared to the aforementioned strains. These findings demonstrate that strain R5 had the ability to produce 2-PE and displayed a great tolerance to 2-PE.

## 4. Conclusions

(1) In this study, a yeast strain was isolated and screened from pear peels that was capable of producing 2-PE. Based on morphology observation, analysis of physiological characteristics, and molecular biological identification, strain R5 was identified as *S. bacillaris*.
(2) Subsequently, through the analysis of whole-genome sequencing and comparison with the KEGG database, we found that strain R5 possesses a metabolic pathway producing 2-PE. To further investigate the synthesis pathway of 2-PE, strain R5 was inoculated into three M3 media. When strain R5 was cultured in M3 (Phe) medium, 2-PE was biosynthesized from L-phe via the Ehrlich pathway. When strain R5 was inoculated in M3 (NH_4_^+^) medium, 2-PE was produced from glucose through the shikimate pathway. In M3 (Phe) medium, the maximum concentration of 2-PE reached 1.28 g/L, which was 16-fold and 2.29-fold higher than that in M3 (NH_4_^+^) and M3 (Phe + NH_4_^+^) media, respectively. These results show that the Ehrlich pathway is the main synthetic pathway for producing 2-PE in strain R5.
(3) The qRT-PCR results revealed that the transport of L-phe was inhibited when both NH_4_^+^ and Phe were present in the medium. The key gene catalyzing the dehydrogenation of benzaldehyde into 2-PE is *ADH5*. And genes *ADH5*, *PDC*, *hisC*, and *GOT1* are not sensitive to nitrogen metabolism inhibition. These findings provide evidence that strain R5 is capable of biotransforming L-phe into 2-PE.
(4) In summary, all the above results illustrated that the Ehrlich pathway and shikimate pathway synthesize 2-PE in strain R5, mainly for the Ehrlich pathway, which had not been previously investigated. These findings fill the research gap relating to the synthesis pathway of 2-PE in strain R5 and provide a solid basis for future research on the application of non-*Saccharomyces* yeasts in the wine industry.

## 5. Accession Number

Details of the accession number are as follows:

SAMN33275642: R5 (TaxID: 1247836)

PRJNA934414: Genome assembly of *Starmerella bacillaris*

The number of Nanopore is SRR23448007, and the number of Illumina is SRR23448008.

## Figures and Tables

**Table 1:** Composition of M3 (Phe), M3 (NH_4_^+^), and M3 (NH_4_^+^ + Phe) media.

	Glucose (g/L)	Sucrose (g/L)	YNB (g/L)	L-phe (g/L)	(NH4)_2_SO_4_ (g/L)	MgSO_4_·7H_2_O (g/L)
M3 (Phe)	30	8	1.7	9	-	0.5
M3 (Phe + NH_4_^+^)	30	8	1.7	4.5	2.25	0.5
M3 (NH_4_^+^)	30	8	1.7	-	4.5	0.5

**Table 2:** Primers of related genes used in this study.

Primers	Sequences
ENO F	5′-TGCTATTGACGCTGCTGGTTA-3′
ENO R	5′-GCCTTGGACTTGTCGGAGTTA-3′
YAT F	5′-GGCGAGGTAGCAGTTAGATTC-3′
YAT R	5′-TGCGGCGGTCAATTCCAA-3′
GOT1 F	5′-TCACCAGTCAACTCGCTTACC-3′
GOT1 R	5′-CAATTTACCACGCAGGGCTTT-3′
hisC F	5′-GCACCTTCAATGGCACCACTA-3′
hisC R	5′-GTCAGCAGTAAGAACGGAGA-3′
PDC F	5′-GGTGTCGTTGATGAGGTTGAGA-3′
PDC R	5′-GGAAGCGTAGAAGGCGTGAA-3′
ADH5 F	5′-TGGCTCAACCTATCAAGTGTCA-3′
ADH5 R	5′-ACCAAGAACAGAAGGCGTGAA-3′

**Table 3:** Identification results of physiology and biochemistry for strain R5.

Items	Results	Items	Results
D-pine trisaccharides assimilate	−	L-lysine arylamase	−
L-malic acid assimilate	+	D-sorbitol assimilate	+
2-Keto-gluconate assimilate	−	L-rhamnose assimilate	−
Glucuronic acid assimilate	+	Xylitol	−
Erythritol assimilate	−	D-sorbitol assimilate	+
Glycerol assimilate	−	Sucrose assimilate	+
D-brown sugar assimilate	−	Urease	−
β-N-acetyl glucosaminidase	+	α-glucosidase	−
Myric acid assimilate	+	Tyrosine arylamase	−
Amygdalin assimilate	−	D-trehalose assimilate	+
α-galactose assimilate	+	Nitrate assimilate	−
Gentiobiose assimilate	+	L-arabinose assimilate	+
D-glucose assimilate	+	Lactose assimilate	+
D-galacturaldehyde assimilate	+	Aesculin hydrolysis	+
Methyl glucoside assimilate	−	L-Glutamate assimilate	+
D-cellobiose assimilate	+	Xylose assimilate	−
γ-glutamyl transferase	−	DL-lactate assimilate	−
D-maltose assimilate	+	Acetate assimilate	−
D-raffinose assimilate	−	Citrate assimilate	−
PNP-n-acetyl-BD-galactosidase	−	Arginine	+
D-mannose assimilate	+	L-proline assimilate	−
D-melibiose assimilate	−	Leucine arylamase	−
N-Acetyl-Glucosamine assimilate	−	D-gluconate assimilate	−

**Table 4:** The whole-genome sequencing of strain R5.

Assembly Feature	R5
Assembled sequence (bp)	9,474,513
No. of scaffolds	250
Sequence depth	204.03
Maximum contig length (bp)	4,175,501
N50 length (bp)	3,806,193
N90 length (bp)	1,100,691
GC content in genome (%)	39.62

**Table 5:** Comparison of the genomes of strain R5, *S. bacillaris* PAS13, and *S. cerevisiae* S288c.

Strain	*S. bacillaris* R5	*S. bacillaris* PAS13	*S. cerevisiae* S288c
Ploidy	n	N	n
Genome size (Mb)	9.47	9.4	12.3
GC content in genome (%)	39.62	39.45	38.3
Total number of CDS	3991	4321	5769

## Data Availability

Not applicable.

## Supplementary Materials

The following are available online at https://www.mdpi.com/article/10.3390/jof9090878/s1, Table S1. Key genes of shikimate pathway and Ehrlich pathway in strain R5.
