# Supplementary material for: Isolation and Identification of Non-Saccharomyces Yeast Producing 2-Phenylethanol and Study of the Ehrlich Pathway and Shikimate Pathway
Source: J Fungi (Basel). 2023 Aug 26;9(9):878. doi: 10.3390/jof9090878 (PMC10532961; doi:10.3390/jof9090878)
Supplement: Supplementary file 1 [file jof-09-00878-s001.zip › jof-2534783-supplementary.pdf]

Table S1. Key genes of shikimate pathway and Ehrlich pathway in strain R5

Number	KO ID	EC ID	KEGG Gene Name	Definition	R5 Genome ID
1	K13853	2.5.1.54 5.4.99.5	aroG, aroA	3-deoxy-7-phosphoheptulonate synthase / chorismate mutase	Contig 1.1159
2	K13830	4.2.3.4 4.2.1.10 1.1.1.25 2.7.1.71 2.5.1.19	Aro1	pentafunctional AROM polypeptide	Contig 2.434
3	K13830	4.2.3.4 4.2.1.10 1.1.1.25 2.7.1.71 2.5.1.19	Aro1	pentafunctional AROM polypeptide	Contig 2.434
4	K00014	1.1.1.25	aroE	shikimate dehydrogenase	Contig3.60
5	K13830	4.2.3.4 4.2.1.10 1.1.1.25 2.7.1.71 2.5.1.19	Aro1	pentafunctional AROM polypeptide	Contig 2.434
6	K13830	4.2.3.4 4.2.1.10 1.1.1.25 2.7.1.71 2.5.1.19	Aro1	pentafunctional AROM polypeptide	Contig 2.434
7	K01736	4.2.3.5	aroC	chorismate synthas	Contig 4.109
8	K06208	5.4.99.5	aroH	chorismate mutase	Contig 1.656
	K01626	2.5.1.54	aroF, aroG, aroH	3-deoxy-7-phosphoheptulonate synthase	Contig 1.176
9	K04518	4.2.1.51	pheA2	prephenate dehydratase	Contig 1.1024
10	K16261		YAT	yeast amino acid transporter	Contig 1.840
	K14454	2.6.1.1	GOT1	aspartate aminotransferase, cytoplasmic	Contig 2.1565
11	K01568	4.1.1.1	PDC	pyruvate decarboxylase	Contig 2.377
12	K00121	1.1.1.284 1.1.1.1	frmA, ADH5 adhC	S-(hydroxymethyl)glutathione dehydrogenase/alcohol dehydrogenase	Contig 2.542
